# Research on the mechanism of prednisone in the treatment of ITP via VIP/PACAP-mediated intestinal immune dysfunction

**DOI:** 10.1186/s40001-023-00987-x

**Published:** 2023-02-08

**Authors:** Xiang Yan, Yayue Zhang, Haiyan Lang, Ziming Huang, Xinyi Chen, Hao He, Qian Zhao, Jun Wang

**Affiliations:** 1 The First Affiliated Hospital of Zhejiang Chinese Medical University (Zhejiang Provincial Hospital of Traditional Chinese Medicine), Hangzhou, China; 2 Dongzhimen Hospital, Beijing University of Chinese Medicine, Beijing, China; 3 Xi’an Medical University, Xi An, Shaanxi China; 4 Department of Hematology, Zhejiang Hospital of Traditional Chinese Medicine, Hangzhou, Zhejiang China

**Keywords:** Immune thrombocytopenia, Vasoactive intestinal peptide, Pituitary adenylate cyclase activating polypeptide, Intestinal immunity, Prednisone

## Abstract

**Rationale:**

Immune thrombocytopenia (ITP) is thought to be a result of immune dysfunction, which is treated by glucocorticoids such as prednisone. Vasoactive intestinal peptide (VIP) and pituitary adenylate cyclase activating polypeptide (PACAP) have immunomodulatory properties, but their role in intestinal immune control is unclear. The major goal of this study was to look at the effects of prednisone on platelet, VIP, and PACAP levels in ITP mice, as well as the regulatory system that controls intestinal immunity.

**Methods:**

Eighteen BALB/c mice were randomly divided into three groups: blank control group, model control group, and prednisone group, with six mice in each group. The ITP animal model control group and the prednisone group were injected with anti-platelet serum (APS) to replicate the ITP animal model. The prednisone group began prednisone intervention on the 8th day. Platelet count was dynamically measured before APS injection, on the 4th day of injection, on the 1st day of administration, on the 4th day of administration, and at the end of the experiment. After the experiment, the expression of p53 protein in mouse mesenteric lymph node lymphocytes was detected by immunohistochemistry. The changes in lymphocyte apoptosis rate in mouse mesenteric lymph nodes were detected by in situ terminal transferase labeling (TUNEL). The contents of VIP and PACAP in the mouse brain, colon, and serum were detected by enzyme-linked immunosorbent assay (ELISA). The contents of IFN-γ, IL-4, IL-10, IL-17A in the mouse spleen were detected by ELISA.

**Results:**

①Changes of peripheral platelet count: there was no significant difference in platelet count among the three groups before modeling; on the 4th day, the platelet count decreased in the model control group and prednisone group; on the 8th day, the number of platelets in model control group and prednisone group was at the lowest level; on the 12th day, the platelet count in prednisone group recovered significantly; on the 15th day, the platelet count in prednisone group continued to rise. ②Changes of VIP, PACAP: compared with the blank control group, VIP and PACAP in the model control group decreased significantly in the brain, colon, and serum. Compared with the model control group, the levels of VIP and PACAP in the brain, colon, and serum in the prednisone group were increased except for serum PACAP. ③Changes of mesenteric lymphocytes: the expression of p53 protein in the mesenteric lymph nodes of model control group mice was significantly higher than that of blank control group mice. After prednisone intervention, the expression of p53 protein decreased significantly.④Changes of cytokines in spleen: compared with blank control group, IFN- γ, IL-17A increased and IL-4 and IL-10 decreased in model control group. After prednisone intervention, IFN- γ, IL-17A was down-regulated and IL-4 and IL-10 were upregulated.

**Conclusions:**

Prednisone-upregulated VIP and PACAP levels decreased P53 protein expression and apoptosis rate in mesenteric lymph node lymphocytes and affected cytokine expression in ITP model mice. Therefore, we speculate that the regulation of intestinal immune function may be a potential mechanism of prednisone in treating ITP.

## Introduction

Immune thrombocytopenia (ITP) is an autoimmune disease characterized by decreased platelet count due to enhanced platelet clearance and impaired platelet production. According to the guidelines issued by the American Society of Hematology (ASH) on adult ITP, the annual incidence of ITP in adults is approximately 2 to 5/100, 000 in the general population [1]. The pathogenesis is not yet fully understood. It is currently believed that the occurrence of ITP is due to the excessive destruction of platelets mediated by humoral and cellular immunity, as well as the abnormal quantity and quality of megakaryocytes, resulting in insufficient platelet production [2, 3]. As the first-line treatment of ITP, prednisone has a significant effect in clinical treatment [1].

Based on the previous research of our research team, the hemostatic mechanism of prednisone in the treatment of ITP is related to the regulation of the levels of vasoactive substances such as vasoactive intestinal peptide (VIP) and 5-hydroxytryptamine (5-HT) [4, 5]. Among them, VIP, as an important neurotransmitter in the brain–gut axis, is called vasodilatory intestinal peptide because of its strong vasodilator effect, which has the functions of dilating blood vessels, transmitting information, and regulating immunity [6]. Pituitary adenylate cyclase activating polypeptide (PACAP), similar to VIP, is also a brain–gut peptide that can act as a neurotransmitter or regulatory mediator released by central or intestinal peptidergic nerves to regulate the physiological role of tissues or organs [7]. In recent years, with the in-depth study of the brain–gut axis theory, more and more studies have confirmed that VIP/PACAP is involved in the regulation of immune function [8, 9]. 70–80% of the immune cells in the human body are distributed in the intestinal tract, and the immune cells in the intestinal lymph nodes secrete signaling molecules such as proteases, histamine, serotonin, and cytokines [10]. Gut-associated lymphoid tissue (GALT) is an intestinal lymphoid tissue and lymphocyte aggregation area involved in immune response initiation and immune tolerance induction. The lamina propria lymphocyte (LPL) diffuses in the lamina propria of the intestinal mucosa, and most of them are CD4 + T cells, including Th1, Th2, Th17, and Treg cells [11, 12].

Therefore, we propose the hypothesis that the mechanism of prednisone treatment of ITP may be related to vasoactive substances and their mediated intestinal immunity. Among them, P53 protein is a transcription factor, mainly distributed in the nucleus, can regulate cell proliferation cycle, differentiation, apoptosis, etc. Through a variety of ways, and its expression can indicate cell apoptosis [13]. Gamma interferon (IFN-γ) is a Th1-related cytokine; interleukin-4 (IL-4) and interleukin-10 (IL-10) are Th2-related cytokines; interleukin-17A (IL-17A) is a Th17-related cytokine [14, 15]. In order to test our hypothesis, we established an ITP model mouse, taking VIP and PACAP-mediated intestinal immune regulation as the main line, and observed the correlation between the occurrence of ITP and the expression levels of VIP and PACAP, as well as other intestinal immune disorders, and detected the mesenteric lymph nodes of mice. The expression of lymphocyte P53 protein, the changes of lymphocyte apoptosis rate, and the contents of VIP and PACAP in different tissues, IFN-γ, IL-4, IL-10, and IL-17A levels to explore the potential mechanism of prednisone in the treatment of ITP.

## Materials

### Animals

SPF grade BALB/c mice, 18, half male and half female, 18–22 g in weight, 8 weeks old, purchased from the Experimental Animal Center of Air Force Military Medical University, experimental animal license number: SCXK (Shaanxi) 2014-002, SPF grade Laboratory IVC mice were housed in cages. Guinea pigs, 10, 3-month-old, female, weighing 250 g, were purchased from Tianrui Experimental Animal Farm in Xingping City, license number SCXK (Shaanxi) 2012-001, and raised in an ordinary environment. This research was approved by the ethics committee of Xi'an Medical College, Shaanxi Province: XYLS2019069.

### Drug

Prednisone (Shanghai Aladdin Biochemical Technology Co., Ltd., specification: 1 g, batch number: 28778).

### Main reagents and instruments

Complete Freund’s adjuvant (Sigma-Aldrich, USA, batch number: SLBF2619V), incomplete Freund’s adjuvant (Sigma-Aldrich, USA, batch number: SLBH7317V), PBS phosphate buffered saline (Beijing Zhongshan Jinqiao Biotechnology Co., Ltd., batch number: ZLI-9062); mouse vasoactive intestinal peptide (VIP) ELISA detection kit (Shanghai Enzyme Link, batch number: ml001911); mouse pituitary adenylate cyclase activating peptide (PACAP) ELISA detection kit (Shanghai Enzyme IL-4 ELISA Kit (Proteintech, USA, Lot: KE10010), Mouse IL-10 ELISA Kit (Proteintech, USA, Lot:KE10010) ELISA detection kit (Proteintech, USA, lot number: KE10008), mouse IL-17A ELISA detection kit (Proteintech, USA, lot number: KE10020). DAPI staining solution (Beijing Leigen Biological Co., Ltd., batch number DA0001); rabbit anti-P53 polyclonal antibody (ABclonal, batch number A3185); goat anti-rabbit working solution (Beijing Zhongshan Jinqiao Biological Co., Ltd., batch number SP-9001); concentrated type DAB kit (Beijing Zhongshan Jinqiao Biological Co., Ltd., batch number K135925C); TUNEL kit (Roche Group, Switzerland, batch number 11684795910). Microcentrifuge (Hunan Changsha Xiangyi Testing Equipment Co., Ltd., model: H1650-W); full-wavelength microplate reader (Thermo Scientific, USA, model: Multiskan GO no. 51119300); automatic animal blood analysis instrument (Beijing Pulang New Technology Co., Ltd., model: XFA6130). TSJ-II Automatic Closed Tissue Dehydrator (Changzhou Zhongwei Electronic Instrument Co., Ltd., China); BMJ-III Embedding Machine (Zhongwei Electronic Instrument Factory, Changzhou Suburbs, China); PHY-III Pathological Tissue Bleaching and Drying Instrument (Changzhou Zhongwei Electronic Instrument Co., Ltd., China); Pannoramic 250 digital slice scanner (Jinan Tangier Electronics Co., Ltd., China); TDZ4-WS desktop low-speed centrifuge (Changsha Xiangyi Centrifuge Instrument Co., Ltd., China); BA400 Model digital trinocular camera microscope (Mike Audi Industrial Group Co., Ltd.); Multiskan GO full-wavelength microplate reader (Thermo Fisher Company, USA); Cytoflex flow cytometer (Beckman Company, USA).

## Methods

### Grouping and model preparation

Before the experiment, blood was collected from the tail vein of 18 BALB/c mice, and the platelet count was detected by an automatic blood cell counter. According to the platelet count, the mice were randomly divided into three groups: the blank control group, the model control group, and the prednisone group, with six mice in each group. Except for the blank control group, mice in other groups were injected with guinea pig anti-mouse platelet serum APS to establish an immune ITP mouse model. The steps were as follows: The BALB/c mice were anesthetized to obtain anticoagulated whole blood, and the platelets were obtained by gradient centrifugation. Adjust the concentration to 2.5 × 10^6^/mL, mix it with equal amounts of complete Freund's adjuvant and incomplete Freund's adjuvant to make antigen, and inject antigen containing complete Freund's adjuvant into the paw, back, and subcutaneous of the guinea pig’s paw at 0 weeks. The antigen containing incomplete Freund's adjuvant was injected at the same site and point as above at 1, 2, and 4 weeks, respectively. In the 5th week, non-anticoagulated whole blood was taken from the guinea pig heart, and the supernatant was centrifuged to obtain the guinea pig anti-mouse platelet serum (GP-APS). Complement was inactivated in a water bath at 56 °C, erythrocytes were adsorbed, and finally diluted with normal saline to a concentration of 1:4 APS for use. Then, except for the blank control group, which was injected with 100 μl/20 g of normal saline, the other groups were injected with APS at a dose of 100 μl/20 g every other day, and the injection was repeated until the end of the experiment, a total of 8 injections. The success of the modeling was determined based on the criteria for identifying the disease-combined model in the National Key Basic Research and Development Program [16].

### Method of administration

The drug administration was started 8 days after the modeling, and each group was given a volume of 0. 1 mL/10 g of drugs for intervention, once per day, for a total of 8 days. The blank control group and the model control group were given normal saline, and the prednisone group was given 2 mg/ml of prednisone.

### Detection indicators and methods

Before the injection of APS, on the 4th day, the 8th day after the injection (the prednisone group started administration), the 12th day, and the 15th day, blood was collected from the tail vein of 18 mice, respectively. Platelet count was determined using a blood analyzer.

The ELISA method detects the content of VIP and PACAP in brain tissue, colon, and serum. A. Specimen preparation after the experiment, the mice in each group were sacrificed, and the brain, colon tissue, and abdominal aortic blood were collected, and the brain and colon tissue were washed with PBS. The brain and colon tissue were ground and homogenized by adding pre-cooled PBS in 9 ratios, centrifuged at 4 °C, 5000 rpm, for 15 min, and the supernatant was collected. If a precipitate forms during storage, centrifuge again to collect the supernatant. B. Detection method according to the instruction manual of the “Mice Vasoactive Intestinal Peptide (VIP) ELISA Detection Kit” and the “Mice Pituitary Adenylate Cyclase Activating Peptide (PACAP) ELISA Detection Kit”, the double-antibody sandwich method was applied. The levels of VIP and PACAP in the mouse brain tissue, colon, and serum were determined. Coat the microplate with purified mouse VIP and PACAP antibodies to make solid-phase antibodies. Add VIP and PACAP to the microwells coated with monoclonal antibody in turn, and then combine them with HRP-labeled VIP and PACAP antibodies to form antibody–antigen–enzyme-labeled antibody complex. After thorough washing, add substrate TMB to develop color. TMB is converted to blue under the catalysis of HRP enzyme and to the final yellow under the action of acid. The depth of color is positively correlated with the level of VIP and PACAP in the sample. The absorbance (OD value) was measured with a microplate reader at a wavelength of 450 nm, and the concentrations of VIP and PACAP in the mouse brain tissue, colon, and serum in the samples were calculated using the standard curve.

Detection of P53 protein expression in mesenteric lymph node lymphocytes by immunohistochemistry Mesenteric lymph node tissue was isolated from mice after dissection and fixed with 4% paraformaldehyde. The fixed tissue was dehydrated by an automatic dehydrator, embedded, sliced, and dewaxed; after three washes in PBS, citrate buffer was added for antigen retrieval, and then goat serum was added dropwise for blocking for 20 min; primary antibody was added dropwise overnight at 4 °C; add biotinylated secondary antibody dropwise, let stand at a 37 °C incubator for 30 min; wash 3 times with PBS, add DAB to develop color; rinse with distilled water, lightly counterstain with hematoxylin, dehydrate, and seal with neutral gum. The images were collected under a microscope, the optical density and area of the images were measured, and the average optical density of each image was calculated.

Determination of the lymphocyte apoptosis rate of mesenteric lymph nodes by the TUNEL method. Lymphoid tissue sections were routinely deparaffinized to water, microwaved with citric acid for 8 min, and then washed with PBS 3 times; a fluorescent Tunel incubation solution (A:B = 1:30) was prepared in the dark. Incubate at 37 °C for 1 h; after washing 3 times with PBS, add DAPI for nuclei staining for 15 min; after washing with PBS, mount the slides with glycerol gelatin, and store at -20 °C; finally, use a digital slice scanner to collect images, and measure the optical density and area of the image to calculate the Apoptosis percentage.

Detection of lymphocyte-related cytokine expression in the spleen by ELISA method. A. Preparation of specimens. After the experiment, mice in each group were sacrificed, spleen tissue was taken, spleen tissue was washed with PBS, and pre-cooled PBS was added at a ratio of 1:9 to grind and homogenize spleen tissue at 4 °C, 5000 rpm, for 15 min, to collect the supernatant of spleen tissue. If a precipitate forms during storage, centrifuge again to collect the supernatant. B. Detection method according to the instruction manual for the “mouse IFN-γ ELISA detection kit", "mouse IL-4 ELISA detection kit”, “mouse IL-10 ELISA detection kit", and "mouse IL-17A ELISA detection kit” The double-antibody sandwich method was used to determine the levels of IFN-, IL-4, IL-10, and IL-17A in the spleen of mice in the sample. Coat the microplates with purified mouse IFN-γ, IL-4, IL-10, and IL-17A antibodies to prepare solid-phase antibodies, and add IFN-γ and IL-4 to the monoclonal antibody-coated microwells in turn, IL-10, IL-17A, and then combined with HRP-labeled IFN-γ, IL-4, IL-10, and IL-17A antibodies to form antibody–antigen–enzyme-labeled antibody complexes. After thorough washing, substrates were added with TMB color. TMB is converted to blue under the catalysis of the HRP enzyme and then to yellow under the action of acid.The shade of color is positively correlated with the levels of IFN-γ, IL-4, IL-10, and IL-17A in the samples. The absorbance (OD value) was measured with a microplate reader at a wavelength of 450 nm, and the protein concentrations of IFN-γ, IL-4, IL-10, and IL-17A in the mouse spleen were calculated by the standard curve.

### Statistical methods

The experimental results were expressed as ($\overline{x}$ ± s). The comparison between groups was performed by one-way ANOVA, and the pairwise comparison was performed by the LSD test. It was considered statistically significant when *P* < 0.05.

## Results

### Effects on the general condition of mice

The mice in the blank control group responded flexibly, with bright hair and no bleeding spots; on the 4th day after APS injection in the model control group and the prednisone group, the mice in the model control group and the prednisone group were sluggish in response, with dry fur and significantly weakened activity. On the 8th day of the experiment, the body weights of the model control group and the prednisone animals were lower than those of the blank control group, and symptoms such as decreased dietary intake and skin bleeding were observed. On the 15th day of the experiment, after drug intervention in the prednisone group, the diet and body weight of the mice increased significantly, the coat color returned to bright, and the bleeding spots were significantly reduced.

### Peripheral platelet count

The platelet counts of mice in each group before APS injection, on the 4th day of injection, on the 1st day of administration, on the 4th day of administration, and at the end of the experiment are shown in Table 1.

**Table 1:** Peripheral platelet count for each group (× 10^9^/L, $\overline{x}$ ± s)

Group	Before treatment	The 4th day	The 8th day	The 12th day	The 15th day
Blank control group	366.83 ± 50.67	379.67 ± 39.40	361.17 ± 43.75	371.83 ± 45.30	357.50 ± 44.02
Model control group	367.83 ± 46.42	228.50 ± 51.64**	175.33 ± 35.60**	179.67 ± 61.55**	181.33 ± 47.56**
Prednisone group	367.50 ± 48.22	229.17 ± 40.31**	178.00 ± 50.65**	260.00 ± 45.59**^△△^	288.33 ± 40.37*^△△^

Compared with the blank control group, *P < 0.05, **P < 0.01; compared with the model group, ^△△^*P* < 0.01

It can be concluded from Table 1: ① before the APS injection, there was no statistical difference in platelet count among the three groups (*P* > 0.05). ② On the 4th day of the experiment, the platelet count in the model control group and the prednisone group decreased, and there was a statistical difference compared with the blank control group (*P* < 0. 01). ③ On the 8th day of the experiment (the first day of administration), the platelet count in the model control group and the prednisone group was at the lowest level, and there was a statistical difference compared with the blank control group (*P* < 0.01). ④ On the 12th day of the experiment (the 4th day of administration), the platelet count in the model control group and the prednisone group was significantly different from that in the blank control group (*P* < 0.01). ⑤ At the end of the experiment (the 8th day of administration), the platelet count in the model control group was significantly different from that in the blank control group (*P* < 0.01); the platelet count in the prednisone group increased significantly, and there was a statistical difference compared with the model control group (*P* < 0.01).

### Brain, colon, serum VIP/PACAP

**Fig. 1:**
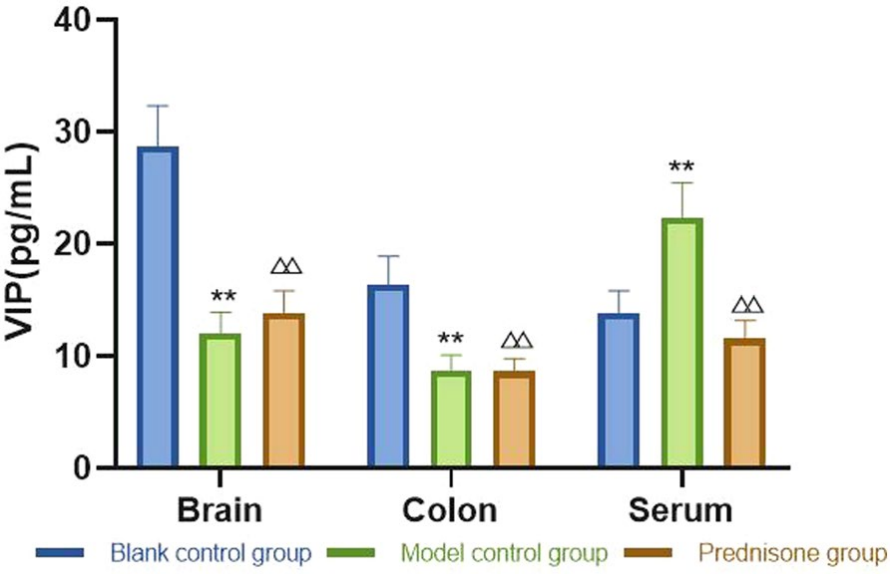
VIP: compared with the blank control group, the VIP levels in the mouse brain, colon, and serum were significantly decreased. Compared with the model control group, the levels of VIP in the prednisone group were increased in brain, colon, and serum. Compared with the blank control group, **P < 0.01; compared with the model group, ^△△^*P* < 0.01

**Fig. 2:**
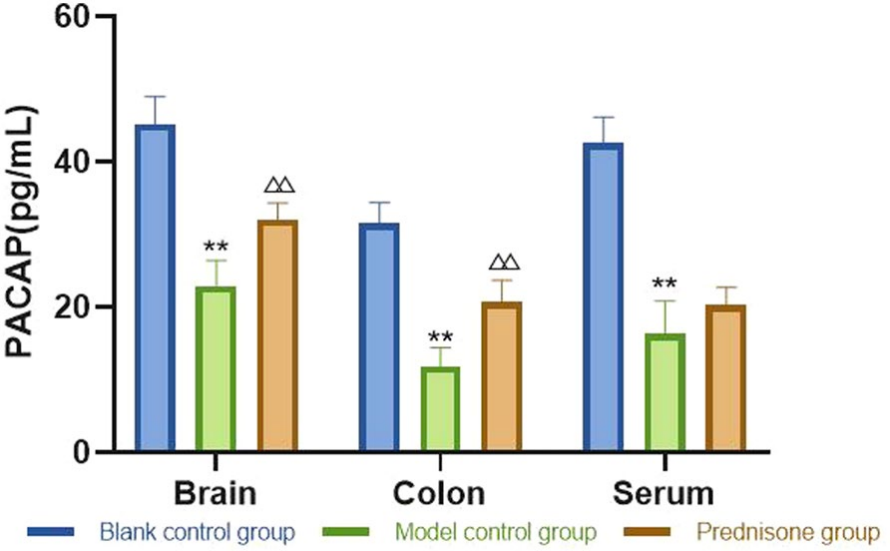
PACAP: compared with the blank control group, the levels of PACAP in the mouse brain, colon, and serum were significantly decreased. Compared with the model control group, the levels of PACAP in the prednisone group were increased in the brain and colon, except in the serum. Compared with the blank control group, **P < 0.01; compared with the model group, ^△△^*P* < 0.01

After the experiment, ELISA was used to detect the contents of VIP and PACAP in the brain, colon, and serum of mice, respectively. The detection results are shown in Figs. 1, 2.

It can be concluded from Figs. 1, 2 that compared with the blank control group, the levels of VIP and PACAP in the model control group were significantly decreased (*P* < 0.05) in the mouse brain, colon, and serum. Compared with the model control group, the levels of VIP in the prednisone group were increased in the brain, colon, and serum (*P* < 0.01); the levels of PACAP in the prednisone group were increased in the brain and colon, except for the serum (*P* < 0.01).

### Mesenteric lymph node lymphocytes

**Fig. 3:**
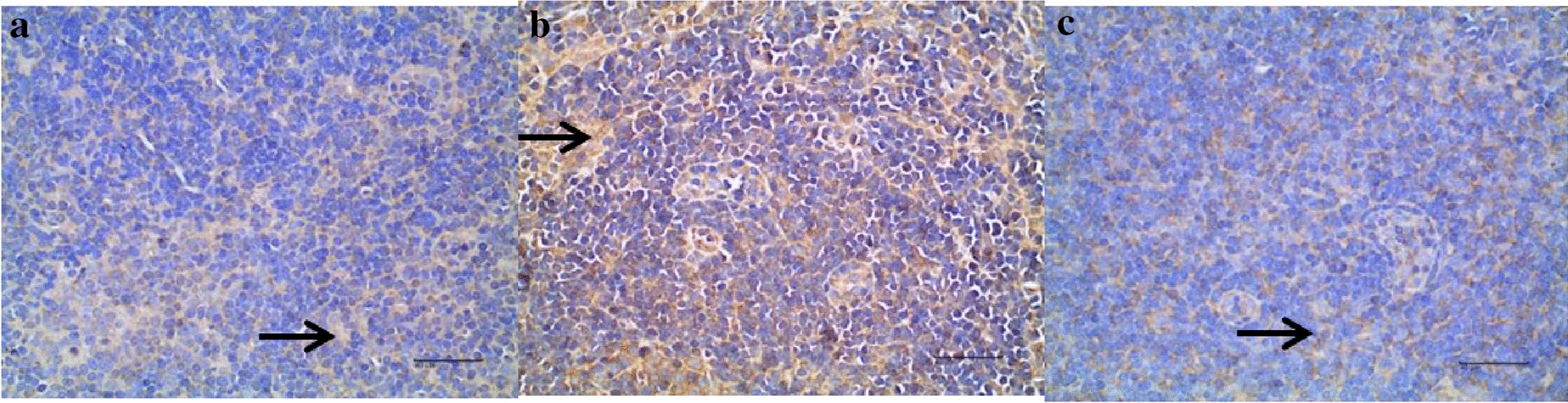
P53 protein expression in each group (× 400). **a** Blank control group: the microscopic images showed that the negative cells were blue, the substrate was white, and the positive cells were yellow or brownish yellow (indicated by black arrows). P53-positive products were mainly distributed in the nucleus, cytoplasm, and intercellular substances. The positive products in the blank control group expressed less. **b** Model control group: the microscopic images showed that the negative cells were blue, the substrate was white, and the positive cells were yellow or brownish yellow (indicated by black arrows). P53-positive products were mainly distributed in the nucleus, cytoplasm, and intercellular substances. The expression of positive products in the model control group was higher than that in the blank control group. **c** Prednisone group: the microscopic images showed that negative cells were blue, substrate was white, positive cells were yellow or brownish yellow (indicated by black arrows), and P53-positive products were mainly distributed in nucleus, cytoplasm, and intercellular substance. The positive products in the prednisone group were less than those in the model control group

**Fig. 4:**
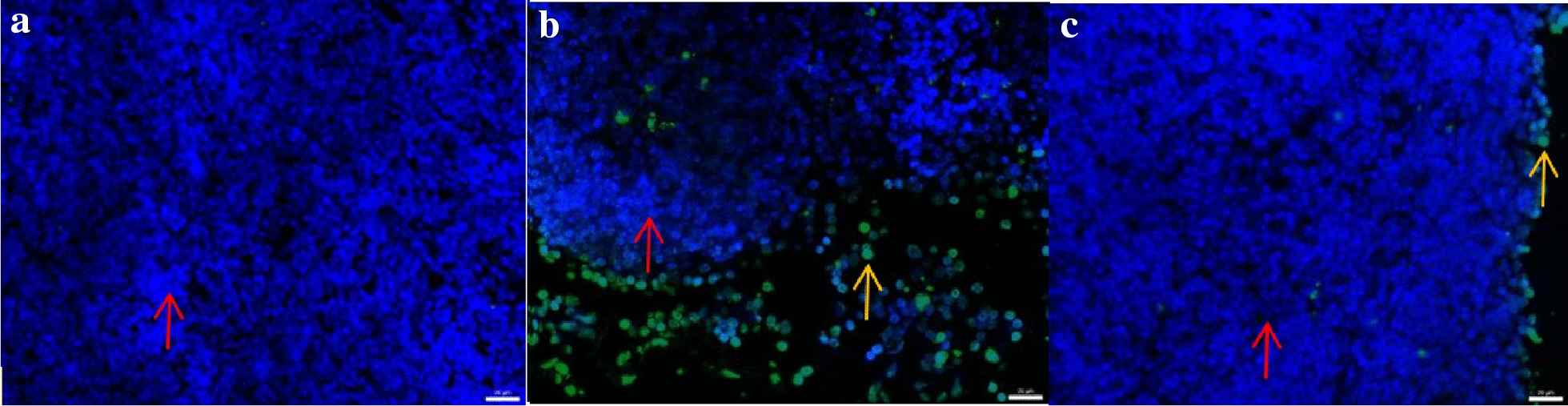
Apoptosis rate of lymphocytes in each group (%, × 400). **a** In the image of the blank control group, the nuclei of apoptotic cells showed green light, and the nuclei of normal cells showed blue light. There were almost no apoptotic cells in the blank control group. Normal cells (↑). **b** Model control group In the image, apoptotic cells show green light, and normal cell nuclei show blue light. A certain number of apoptotic cells were seen in the model control group. Normal cells (↑) and apoptotic cells (↑). **c** In the images of the prednisone group, apoptotic cells showed green light, and normal cells showed blue light. A few apoptotic cells were seen in the prednisone group. Normal cells (↑) and apoptotic cells (↑)

**Fig. 5:**
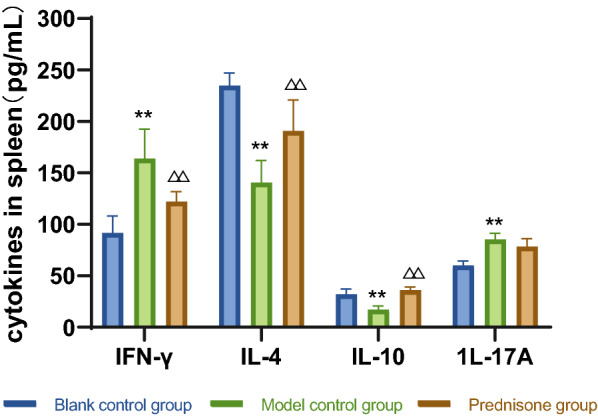
Changes of cytokines in spleen (pg/ml, $\overline{x}$ ± s) The contents of IFN-γ and IL-17A in the spleen of the model control group were increased compared to the blank control group, while the contents of IL-4 and IL-10 were decreased. The level of IFN-γ in the prednisone group was lower than in the model control group, as was the level of IL-17A, and the levels of IL-4 and IL-10 were higher

After the experiment, the mice were dissected and separated from the mesenteric lymph nodes, and the changes in the expression of P53 protein and the apoptosis rate in the lymphocytes of the mesenteric lymph nodes were detected by immunohistochemistry. The comparison of P53 protein expression and the apoptosis rate of mesenteric lymph node lymphocytes in each group of mice is shown in Tables 2, 3 and Figs. 3b, c, 4a–c and 5.

**Table 2:** P53 protein expression in each group ($\overline{x}$ ± s)

Group	N	OD($\overline{x}$ ± s)
Blank control group	6	0. 2089 ± 0.0087
Model control group	6	0. 2515 ± 0.0275**
Prednisone group	6	0. 2187 ± 0.0253^△△^

Compared with the blank control group, *P < 0.05, **P < 0.01; compared with the model control group, ^△△^*P* < 0.01

**Table 3:** Apoptosis rate of lymphocytes in each group (%, $\overline{x}$ ± s)

Group	N	Apoptosis
Blank control group	6	0.67 ± 0.43
Model control group	6	21.69 ± 5.38**
Prednisone group	6	5.56 ± 2.07**^△△^

Compared with the blank control group, *P < 0.05, **P < 0.01; compared with the model control group, ^△△^*P* < 0.01

From Tables 2, 3 and Figs. 3a–c and 4a–c, it can be concluded: ① as shown in Figs. 1, 2, 3, the expression of positive products in the blank control group is lower, the expression of positive products in the model control group is higher, and the positive products in the prednisone group are less than those in the model control group. Table 2 shows that, compared with the blank control group, the expression of P53 protein in the mesenteric lymph node lymphocytes of the mice in the model control group was increased (*P* < 0.01), and the apoptosis rate of lymphocytes was increased (*P* < 0.01). ② As shown in Figs. 3b, c and 4a, apoptotic cells show green light in the image, and normal cell nuclei show blue light. There were almost no apoptotic cells in the blank control group, a certain number of apoptotic cells in the model control group, and a few apoptotic cells in the prednisone group. Table 4 shows that the apoptosis rate of mesenteric lymphocytes in the model control group was significantly higher than that in the blank control group (*P* < 0.01); compared with the model control group, the apoptosis rate of the prednisone group was significantly decreased (*P* < 0.01).

**Table 4:** Cytokines expression in each group (pg/ml, $\overline{x}$ ± s)

Group	Blank control group	Model control group	Prednisone group
IFN-γ	91.63 ± 16.40	163.91 ± 28.74**	122.16 ± 9.69**^△△^
IL-4	234.98 ± 12.03	140.64 ± 21.58C	190.87 ± 30.02**^△△^
IL-10	32.02 ± 5.04	17.09 ± 3.46**	36.04 ± 3.10^△△^
1L-17A	60.04 ± 4.45	85.33 ± 6.11**	78.35 ± 7.82**

Compared with the blank control group, *P < 0.05, **P < 0.01; compared with the model group, ^△△^*P* < 0.01

### Changes of cytokines in spleen

After the experiment, the levels of IFN-γ, IL-4, IL-10 and IL-17A in spleen were detected by ELISA. The test results are shown in Table 4 and Fig. 5.

From Table 4 and Fig. 5, it can be concluded that compared with the blank control group, the contents of IFN-γ and IL-17A in the model control group were increased in the spleen (*P* < 0.01), and the contents of IL-4 and IL-10 in the spleen were decreased (*P* < 0.01). Compared with the model control group, the level of IFN-γ in the prednisone group was down-regulated in the spleen (*P* < 0.01), the level of IL-17A was also decreased, and the levels of IL-4 and IL-10 were increased in the spleen (*P* < 0.01).

## Discussion

The main mechanism of prednisone as a first-line treatment for ITP is to inhibit humoral and cell-mediated immune responses, reduce platelet autoantibody production and mitigate antigen–antibody reactions; inhibit the phagocytosis of the monocyte–macrophage system, reduce platelet destruction, and thus elevate platelets [2]. In this study, we established ITP model mice, and by comparing the blank control group, model control group, and prednisone group, we again confirmed that prednisone could increase platelet count in ITP mice, and platelet count recovered significantly in the prednisone group on day 4 of administration, and continued to increase on day 8 after administration.

VIP is a 28-amino acid neuropeptide/neurotransmitter that is widely distributed in the central and peripheral nervous system [6]. VIP is released by both neuronal and immune cells. Various cell types, including immune cells, express VIP receptors [7]. VIP has pleiotropic effects as a neurotransmitter, immunomodulator, vasodilator, and pro-secretory agent [17]. PACAP is a 38-amino acid neuropeptide in the glucagon superfamily that acts together with glucagon and VIP [18]. The nervous and immune systems are involved in complex bidirectional regulation through neuropeptides such as VIP/PACAP in several organs [19]. In our experimental results, we found that the levels of VIP and PACAP were significantly reduced in ITP model mice compared with normal mice, suggesting that VIP and PACAP may be an important part of the pathogenesis of ITP. And after prednisone intervention, the levels of both increased significantly compared with the model control group, except for serum PACAP, suggesting that prednisone has a modulatory effect on VIP and PACAP.

The intestinal mucosa is the largest immune system in the body and is the largest area in contact with the outside world. The intestine contains the luminal microbiota and a large number of immune cells in the epithelium, lamina propria and lymphoid follicles, including Th1, Th2, Th17 and Treg cells [20]. A new VIP/PACAP immunomodulatory mechanism has now been identified, which expresses low levels of IFN-γ, as well as high levels of IL-10, by inducing tolerant dendritic cells (tDC) to produce antigen-specific regulatory T cells (Treg) [21]. Meanwhile, IL-10 is a key mediator of Bregs, and under appropriate stimulation, Bregs express IL-10 [22, 23]. SUN X et al. [24] demonstrated that VIP plays an important role in the stabilization of IL-10 mRNA in Bregs and effectively suppresses the experimental inflammatory response in mice. In addition, VIP/PACAP, as a regulator involved in inflammation and autoimmune diseases, can directly affect immune cells and regulate Th1/Th2 and Th17/Treg cell imbalances [9, 25, 26].

In our study, there was an abnormal upregulation of P53 protein expression in the mesenteric lymph node lymphocytes of ITP model mice. By comparison, we found that P53 protein expression was significantly higher in the model control group mice compared with the blank control group mice, while P53 expression in lymphocytes was significantly lower after prednisone intervention, suggesting that prednisone can reduce P53 protein expression. Meanwhile, the apoptotic rate of lymphocytes in the mesenteric lymph nodes of ITP model mice was abnormally high, and prednisone intervention also significantly reduced the apoptotic rate of lymphocytes. This confirmed that prednisone could improve intestinal immunity and reduce lymphocyte apoptosis. Meanwhile, by analyzing the changes of related cytokine levels in the experiment, it was found that IFN-γ and IL-17A were elevated and IL-4 and IL-10 were decreased in the spleen of ITP mice in the model control group. And after prednisone intervention, the levels of these cytokines were reversed with the upregulation of VIP/PACAP expression. It was confirmed that prednisone could regulate intestinal immune function and maintain immune homeostasis by affecting VIP and PACAP expression, thus exerting a therapeutic effect on ITP.

## Conclusion

In summary, VIP/PACAP levels in the model control group of ITP mice were decreased in brain, colon, and serum, and P53 protein expression and apoptosis rate in mesenteric lymph node lymphocytes were abnormally increased, and cytokine changes such as elevated IFN-γ, decreased IL-4 and IL-10, and elevated IL-17A were observed. After prednisone intervention, VIP/PACAP levels were elevated in brain, colon, and serum, except for serum PACAP; P53 protein expression and lymphocyte apoptosis rate were reduced, while related cytokine levels were reversed. Therefore, we speculate that VIP/PACAP-based modulation of intestinal immune function may be a potential mechanism for prednisone treatment of ITP. Our experiments again demonstrate that VIP/PACAP is an important part of ITP pathogenesis, and targeted therapy targeting VIP/PACAP-mediated intestinal immune function may become a new research direction for ITP treatment in the near future. Of course, more studies are needed to further explain these mechanisms to this process.

## Data Availability

The datasets used and/or analyzed during the current study are available from the corresponding author on reasonable request.
